# Association of Mental Health-Related Proteins DAXX, DRD3, and DISC1 With the Progression and Prognosis of Chondrosarcoma

**DOI:** 10.3389/fmolb.2019.00134

**Published:** 2019-11-26

**Authors:** Lile He, Xiangyu Shi, Ruiqi Chen, Zhengchun Wu, Zhulin Yang, Zhihong Li

**Affiliations:** ^1^Department of Orthopedics, The Second Xiangya Hospital, Central South University, Changsha, China; ^2^Hunan Key Laboratory of Tumor Models and Individualized Medicine, Changsha, China; ^3^Department of Cardiovascular Medicine, The Second Xiangya Hospital, Central South University, Changsha, China; ^4^Department of General Surgery, The Second Xiangya Hospital, Central South University, Changsha, China

**Keywords:** DAXX, DRD3, DISC1, immunohistochemistry, chondrosarcoma, osteochondroma, mental health

## Abstract

Chondrosarcoma is the second most common malignant bone tumor. Current therapies remain ineffective, resulting in poor prognoses. Biomarkers for chondrosarcoma and predictors of its prognosis have not been established. Mental health–related proteins have been associated with the pathogenesis, progression, and prognosis of many cancers, but their association with chondrosarcoma has not been reported. In this study, the expression and clinicopathological significance of the mental health–related proteins DAXX, DRD3, and DISC1 in chondrosarcoma tissue samples were examined, over an 84-months follow-up period. In immunohistochemical analysis, the rates of positive DAXX, DRD3, and DISC1 expression were significantly higher in chondrosarcoma than in osteochondroma tissue (*P* < 0.01). The percentages of positive DAXX, DRD3, and DISC1 expression were significantly lower in tissues with good differentiation (*P* < 0.01), AJCC stage I/ II (*P* < 0.01), Enneking stage I (*P* < 0.01), and non-metastasis (*P* < 0.05), respectively. In Kaplan–Meier survival analysis, significantly shorter mean survival times were associated with moderate and poor differentiation (*P* = 0.000), AJCC stage III/IV (*P* = 0.000), Enneking stage II/III (*P* = 0.000), metastasis (*P* = 0.019), invasion (*P* = 0.013), and positive DAXX (*P* = 0.012), and/or DRD3 (*P* = 0.018) expression. In Cox regression analysis, moderate and poor differentiation (*P* = 0.006), AJCC stage III/IV (*P* = 0.013), Enneking stage II/III (*P* = 0.016), metastasis (*P* = 0.033), invasion (*P* = 0.011), and positive DAXX (*P* = 0.033), and/or DRD3 (*P* = 0.025) staining correlated negatively with the postoperative survival rate and positively with mortality. In competing-risks regression analysis, differentiation (*P* = 0.005), metastasis (*P* = 0.014), invasion (*P* = 0.028), AJCC stage (*P* = 0.003), Enneking stage (*P* = 0.036), and DAXX (*P* = 0.039), and DRD3(*P* = 0.019) expression were independent predictors of death from chondrosarcoma. The areas under receiver operating characteristic curves for DAXX, DRD3, and DISC1 expression were 0.673 (95% CI, 0.557–0.788; *P* = 0.010), 0.670 (95% CI, 0.556–0.784; *P* = 0.011), and 0.688 (95% CI, 0.573–0.802; *P* = 0.005), respectively. These results suggest that DAXX, DRD3, and DISC1 could serve as biomarkers of chondrosarcoma progression and predictors of its prognosis.

## Introduction

Patients with chondrosarcoma (CS), a cartilage malignancy that accounts for 25% of skeletal system sarcomas (Andreou et al., 2011), often have a poor prognosis (Group, 2014). CS is aggressive, characterized by early metastasis in 42% of patients and local recurrence in up to 86% of patients. Current therapies for CS, including surgical resection, chemotherapy, and radiotherapy, are ineffective. According to recent studies, 5-years survival rates are about 79% for patients with conventional central CS (Fromm et al., 2018), 7–24% for patients with dedifferentiated CS, and 28% for those with mesenchymal CS (MacDonald et al., 2019). Chemotherapy after surgical resection has been proven to have no benefit in CS, with a combined 5-years survival rate of 53% (Peterse et al., 2017). Moreover, due to the limited overall efficacy of chemotherapy, the 5-years survival rate for patients with unresectable CS is only 2% (Peterse et al., 2017). On the other hand, as CS grows slowly, with a relatively low fraction of dividing cells, chondrogenic tumors are considered to be radioresistant, as radiotherapy acts on dividing cells (Gelderblom et al., 2008). These clinical findings emphasize the urgent need for the development of novel effective strategies for the treatment of CS. However, despite great research efforts, no CS biomarker or prognosis predictor has been established for clinical practice.

People with poor mental health are reportedly at greater risk of cancer (Levine et al., 1978; Cohen et al., 2007), and mental health–related proteins, such as death domain–associated protein (DAXX) (Tang et al., 2014), dopamine D3 receptor (DRD3) (Castorina et al., 2010), and disrupted-in-schizophrenia-1 (DISC1) (Gao et al., 2016), have shown significant associations with cancer pathogenesis, progression, and prognosis. But the roles of these proteins in CS have not been studied.

Many studies have examined the roles of DAXX in mental diseases and disorders, including Parkinson's disease (Karunakaran et al., 2007) and schizophrenia (Subburaju et al., 2016). DAXX has multiple functions, such as transcriptional regulation, apoptosis, and carcinogenesis. It was originally identified as a Fas death receptor that binds to apoptosis-associated proteins through Jun N-terminal kinase pathways (Chang et al., 1998). However, study findings are conflicting, and its role remains unclear. DAXX is either anti-apoptotic or pro-apoptotic. It has multiple alternative localizations in the nucleus and cytoplasm, likely associated with different functions. DAXX was found to translocate from the nucleus to the nuclear membrane, cytoplasm, or cell membrane with the progression of cervical cancer (Tang et al., 2014). Alteration of its intracellular distribution range in urinary tract epithelial cells results in urothelial carcinoma (Zizzi et al., 2013). DAXX mutations have been identified in a wide range of tumor types, at a rate of 43%. The first observation was made in pancreatic neuroendocrine tumors (pNETs), which showed frequent mutations in DAXX chromatin factors (Jiao et al., 2011). DAXX mutations were also detected in alternative lengthening of telomeres (ALT) neuroblastoma with chemoresistance and late recurrence, and in other ALT contexts (Heaphy et al., 2011). The loss of DAXX/ATRX expression was observed in some pNET tissue and correlated with chromosome instability, advanced disease, and poor prognosis (Marinoni et al., 2014). Recently, Shi et al. (2019) reported that DAXX is a modulator of DNA damage repair and suppressor of triple negative breast cancer progression. In addition, DAXX may function as a novel anaphase-promoting complex or cyclosome inhibitor and promote chromosome-instability cancer predisposition during prostate cancer development (Kwan et al., 2013). These findings suggest that dislocations or mutations alter the function of DAXX, sometimes contributing to tumor development.

Dopamine receptors are G-protein coupled receptors that respond to the neurotransmitter dopamine. They can be classified into two main groups: D1-like (DRD1 and DRD5) and D2-like (DRD2, DRD3, and DRD4) receptors. Most studies of these receptors have been in the field of neurobiology; their roles in cancer remain unclear. Researchers believe that mental and physiological conditions play important roles in cancer development and progression (Barik et al., 2013); chronic stress has been shown to cause the secretion of neurotransmitters that participate in such activities in several cancers, such as breast cancer (So et al., 2010). Several neurotransmitters cause tumor progression through the stimulation of tumor cell migration and dissemination to distant sites, and through the activation of signaling pathways linked to cell proliferation and survival (e.g., the phosphatidyl inositide 3-kinase, mitogen-activated protein kinase, and Akt pathways) (Beaulieu and Gainetdinov, 2011). Such events occur due to the secretion of catecholamines (stress mediators), which increases in response to stress. The catecholamines epinephrine and norepinephrine are related to carcinogenesis and tumor progression. Recently, nervous system mediators, including neurotransmitters, have also been shown to play important roles in tumor angiogenesis. Dopamine has proliferative effects in non-transformed cells and inhibits T cell receptor–induced cell proliferation, leading to immune system depletion via the stimulation of T-cell cytokine secretion (Basu et al., 2010). It also has an inhibiting effect on breast cancer cells and in several other cancers (Chakroborty et al., 2004).

Data on the role of D2-like receptors in cancer are conflicting. On one hand, D2-like receptor activation has been reported to induce apoptosis or to inhibit cancer cell proliferation (Roy et al., 2017). On the other hand, D2-like receptor antagonism was found to have anticancer effects (Sachlos et al., 2012). Moreover, a study has shown that chronic stress changes D2-like receptor gene expression profiles in non-small cell lung cancer (Sheikhpour et al., 2013). Castorina et al. (2010) found that the DRD3 agonist contributes to the development of malignant peripheral nerve sheath tumors through the inhibition of tumor suppressor gene-NF1 expression. In pancreatic ductal adenocarcinoma (Jandaghi et al., 2016) and glioblastoma (Li et al., 2014), DRD2 knockdown significantly reduced tumor cell viability. In cervical and endometrial cancers, melanoma cells, and breast cancer stem cells, single-agent treatment with D2-like receptor antagonists is antitumorigenic (Kang et al., 2012; Sachlos et al., 2012). Hoeppner et al. (2015) reported that the D2-like receptor agonist inhibits lung cancer progression by reducing angiogenesis and tumor infiltration of myeloid-derived suppressor cells. In gastric cancer cells, D2-like receptor activation can reduce cancer cell proliferation through the upregulation of Krüppel-like factor 4 via inhibition of ERK1/2 and Akt phosphorylation (Ganguly et al., 2010). Thus, whether DRD3 has carcinogenic effects in CS is unknown.

DISC1, associated with a variety of brain disorders, activates Wnt/β-catenin signaling by inhibiting glycogen synthase kinase 3 beta (GSK3β) phosphorylation, and promotes neural progenitor cell proliferation. Aberrations in the GSK3β/β-catenin signaling pathway have been associated with the pathogenesis of liver and lung cancers (Wan et al., 2015; Wang et al., 2017). Gao et al. who first identified DISC1 as an oncogene in glioblastoma tumorigenesis. They observed greater expression of DISC1 in glioma cells than in normal cells, and found that DISC1 knockdown significantly inhibited glioblastoma cell proliferation, migration, invasion, and stem cell self-renewal (Gao et al., 2018). Their studies showed that DISC1 inhibition altered mitochondrial dynamics by regulating Drp1 (Gao et al., 2016), and that Drp1 activation was associated with a poor prognosis of glioblastoma (Xie et al., 2015), indicating that mitochondrial dynamics acted as a regulatory switch for the differentiation of glioma stem cells and could be a new therapeutic target. Drp1 was also found to regulate glioma cell proliferation and invasion via the RHOA/ROCK1 pathway (Yin et al., 2016). DISC1 also regulates the cyclic adenosine monophosphate (cAMP) signaling pathway, which is associated closely with cancer pathogenesis and progression (Simko et al., 2017) via binding and inhibition of phosphodiesterase 4B (PDE4B). All of these studies showed that DISC1 expression is associated with the oncogenesis, progression, and prognosis of cancer, but its role in CS has not been examined.

In the present study, we hypothesized that the expression of mental health–related proteins would be associated with the progression and poor prognosis of CS. Osteochondroma (OC) was used as a control, as it is a benign bone tumor and its malignant degeneration usually causes CS (Somers and Faber, 1999; Baatenburg de Jong et al., 2004; Limaiem and Sticco, 2019). DAXX, DRD3, and DISC1 protein expression in CS and OC was investigated by immunohistochemistry (IHC). The clinical and pathological significance of these proteins in CS, and their roles in the progression and prognosis of this malignancy, were analyzed. To our knowledge, this study is the first to investigate associations between mental health–related protein expression and CS.

## Materials and Methods

### Specimens and Clinical Data

This study was conducted with resected tumor specimens from 80 patients with CS and 25 patients with OC collected between January 2011 and June 2015 at the Second and Third Xiangya hospitals, Central South University, Changsha, China. Diagnoses were confirmed by histopathology. Tissues were formalin fixed and paraffin embedded using standard procedures. Patients with CS or their relatives were contacted by telephone and/or email to obtain survival information. Deaths were confirmed by checking the death records at The Ministry of Public Security of the People‘s Republic of China.

Clinicopathological data collected included patient age and sex, extent of tumor differentiation, tumor size, AJCC and Enneking stages, and the presence of metastasis and/or invasion. Tumor differentiation is a clinical parameter characterizing the degree to which tumor cells resemble those of the tissue from which they arise; high degrees of differentiation imply low malignancy and *vice versa*. The AJCC stage system (I–IV) characterizes the extent and severity of cancer progression, with IV representing the most advanced stage. The Enneking stage system is commonly used for bone cancer; stage I represents low-grade localized tumors, stage II represents high-grade localized tumors, and stage III represents metastatic tumors (regardless of grade). Metastasis is the spread of a pathogenic agent (i.e., tumor) from a primary to a secondary site in the host's body. It is generally distinguished from invasion, which is the direct extension and penetration of cancer cells into neighboring tissues.

### Ethics Statement

This study was approved by the Medical Ethics Committee of the Second Xiangya Hospital, Central South University and performed in accordance with the Declaration of Helsinki. Written informed consent was obtained from each participant or his/her legal custodian.

### Immunohistochemistry

Four-micrometer-thick sections were cut from the paraffin-embedded tissues. IHC staining for DAXX, DRD3, and DISCC1 was performed using the EnVision^TM^ detection kit (Dako Laboratories, Carpinteria, CA, USA) according to the manufacturer's protocol. Briefly, the sections were deparaffinized and then incubated with peroxidase inhibitor (3% H_2_O_2_) in the dark for 15 min, followed by incubation in sodium citrate buffer (10 mM Sodium citrate, 0.05% Tween 20, pH 6.0) at room temperature for 20 min. The tissue sections were then incubated with rabbit anti-human DAXX, DRD3, and DISC1 antibodies (Boster Biological Technology Co., Ltd., California, USA) for 2 h, followed by rinsing with PBS three times for 5 min each. Solution A [containing HRP-conjugated anti-rabbit secondary antibody (Santa Cruz Biotechnology)] was then added to the sections and incubation was continued for 30 min at room temperature. After rinsing with 1× PBS three times for 5 min each, the sections were stained with DAB, counterstained hematoxylin, dehydrated with alcohol, soaked in xylene, and mounted with neutral balsam. Positivity for DAXX, DRD3, or DISC1 was defined as ≥20% positively stained cells at a staining strength ≥2, or 10–20% positivity stained cells at a strength of 3.

### Statistical Analysis

Data were analyzed using the Statistical Package for the Social Sciences version 18.0 (SPSS Inc., Chicago, IL, USA). DAXX, DRD3, and DISC1 expression in CS and OC specimens, and relationships of this expression to histological and clinical factors, were analyzed using the chi-squared test. The Kaplan–Meier and log-rank tests were used for univariable survival analysis, with calculation of receiver operating characteristic (ROC) curves and areas under the curves (AUCs). Cox proportional-hazards and competing-risks regression models proposed by Fine and Gray (1999) were used for multivariable analysis and the determination of 95% confidence intervals (CIs). The latter were used to account for the possibility that patients died of other causes. *P* < 0.05 was considered to represent significance.

Proportional hazards (PH) assumption was evaluated by calculating weighted Schoenfeld residuals, and no violations of the PH assumptions for the DAXX, DRD3, DISC1 in Cox model was found (*P* > 0.05). Assumption for the Chi-square test which require the sample size ≥40 and the expected number of participants ≥5 in each group was check, and all data met the requirement for Chi-square tests.

## Results

### DAXX, DRD3, and DISC1 Protein Expression in CS and OC Tissues

**Figure 1:**
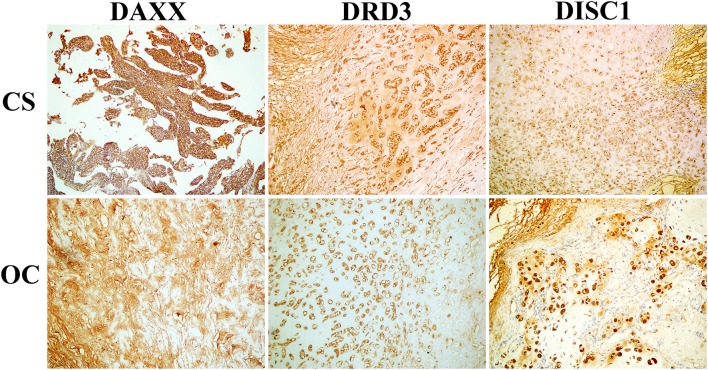
Representative patterns of DAXX, DRD3, and DISC1 expression in chondrosarcoma (CS) and osteochondroma (OC) tissues are shown (×200 original magnification).

Positive DAXX staining was located mainly in the cytoplasm and nuclei, and positive DRD3 and DISC1 staining was located mainly in the cytoplasm. Of the 80 CS samples, positive DAXX, DRD3, and DISC1 staining was observed in 41 (51.3%), 40 (50.0%), and 46 (57.5%) samples, respectively. Of the 25 OC samples, 4 (16.7%) were DAXX positive, 4 (16.0%) were DRD3 positive, and 5 (20.0%) were DISC1 positive. These proportions were significantly greater in CS than in OC samples (*P*_DAXX_ = 0.003, *P*_DRD3_ = 0.003, *P*_DISC1_ = 0.001; Figure 1, Table 1).

**Table 1:** DAXX, DRD3, and DISC1 expression in chondrosarcoma and osteochondroma.

	**Chondrosarcoma**		**Osteochondroma**		
	***n***	**%**	***n***	**%**	**χ^**2**^**	***P***
**DAXX**
–	39	48.8	20	83.3	8.995	0.003
+	41	51.3	4	16.7		
**DRD3**
–	40	50.0	21	84.0	9.045	0.003
+	40	50.0	4	16.0		
**DISC1**
–	34	42.5	20	80.0	10.723	0.001
+	46	57.5	5	20.0		

### Association of DAXX, DRD3, and DISC1 Expression With the Clinicopathological Features of CS

DAXX, DRD3, and DISC1 expression in CS samples was not associated with patient age or sex, tumor size, or invasion (Table 2). The percentages of positive DAXX, DRD3, and DISC1 expression were significantly greater in tissues with moderate and poor differentiation, AJCC stage III/IV, Enneking stage II/III, and metastasis than in those with good differentiation (*P* < 0.01), AJCC stage I/II (*P* < 0.01), Enneking stage I (*P* < 0.01), and no metastasis (*P* < 0.05), respectively.

**Table 2:** Associations of DAXX, DRD3, and DISC1 expression with the clinicopathological characteristics of chondrosarcoma.

**Characteristics**	***n***	**DAXX positive *n* (%)**	**χ^**2**^**	***P***	**DRD3 positive *n* (%)**	**χ^**2**^**	***P***	**DISC1 positive *n* (%)**	**χ^**2**^**	***P***
**AGE (YEARS)**										
<45	34	21 (61.8)	2.617	0.106	19 (55.9)	0.818	0.366	22 (64.7)	1.256	0.262
≥45	46	20 (43.5)			21 (45.7)			24 (51.2)		
**SEX**										
Male	43	21 (48.8)	0.217	0.641	21 (48.8)	0.050	0.823	26 (60.5)	0.334	0.563
Female	37	20 (50.1)			19 (51.4)			20 (54.1)		
**DIFFERENTIATION**										
Good	58	23 (39.7)	11.349	0.000	23 (39.7)	9.028	0.003	25 (43.1)	20.065	0.000
Moderate and Poor	22	18(81.8)			17 (77.3)			21 (95.5)		
**TUMOR SIZE**										
<5 cm	24	11 (45.8)	0.403	0.525	12 (50.0)	0.000	1.000	12 (50.0)	0.789	0.374
≥5 cm	56	30 (53.6)			28 (50.0)			34 (60.7)		
**AJCC STAGE**										
I /II	61	25(41.0)	10.835	0.001	25 (41.0)	8.352	0.004	28 (45.9)	14.138	0.000
III/IV	19	16 (84.2)			15(78.9)			18 (94.7)		
**ENNEKING STAGE**										
I	55	20 (36.4)	15.611	0.000	21 (38.2)	9.833	0.002	23 (41.8)	17.711	0.000
II/ III	25	21 (84.0)			19 (76.0)			23 (92.0)		
**METASTASIS**										
No	69	32 (46.4)	4.770	0.029	31 (44.9)	5.165	0.023	36 (52.2)	5.825	0.016
Yes	11	9 (81.8)			9 (81.8)			10 (90.9)		
**INVASION**										
No	13	7 (53.8)	0.042	0.838	8 (61.5)	0.872	0.363	9 (69.2)	0.874	0.350
Yes	67	34 (50.7)			32 (47.6)			37 (55.2)		

### Correlation of DAXX, DRD3, and DISC1 Expression in CS

Of the 41 CS tissue samples with positive DAXX expression, 34 were DRD3 positive and 34 were DISC1 positive. Of the 39 tissue samples with negative DAXX expression, 33 were DRD3 negative and 27 were DISC1 negative. Of the 40 samples with positive DRD3 expression, 33 were DISC1 positive. Of the 40 samples with negative DRD3 expression, 27 were DISC1 negative. Significant positive correlations were found between the expression of DAXX and DRD3 (χ^2^ = 36.473, *P* = 0.000), DAXX and DISC1 (χ^2^ = 22.25, *P* = 0.000), and DRD3 and DISC1 (χ^2^ = 20.46, *P* < 0.001).

### Correlations of Clinicopathological Parameters and DAXX, DRD3, and DISC1 Expression With the Mean Survival of Patients With CS

**Figure 2:**
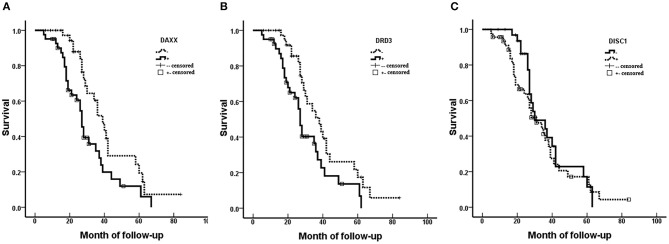
Associations of DAXX, DRD3, and DISC1 expression with survival in patients with chondrosarcoma. Prognoses are depicted with Kaplan–Meier curves. **(A)** DAXX expression (mean survival, positive 30.41 vs. negative 42.13 months; *P* = 0.012). **(B)** DRD3 expression (mean survival, positive 30.87 vs. negative 40.85 months; *P* = 0.018). **(C)** DISC1 expression (mean survival, positive 34.09 vs. negative 37.34 months; *P* = 0.404).

Fifty-three (66.3%) patients died during the follow-up period (maximum, 84 months) and 27 (33.7%) patients (including censored cases) survived. Survival was censored at 84 months. Kaplan–Meier analysis revealed significantly shorter mean survival times in patients with moderately and poorly differentiated tumors (*P* = 0.000), AJCC stage III/IV CS (*P* = 0.000), Enneking stage II/III CS (*P* = 0.000), metastasis (*P* = 0.019), invasion (*P* = 0.013), positive DAXX staining (*P* = 0.012), and positive DRD3 staining (*P* = 0.018). The mean patient survival time was not associated with patient age or sex, tumor size, or DISC1 expression (Figure 2, Table 3).

**Table 3:** Relationships of DAXX, DRD3, and DISC1 expression and clinicopathological characteristics to average survival in patients with chondrosarcoma.

**Clinicopathological characteristics**	***n***	**Average survival (months)**	**χ^**2**^**	***P***
**AGE (YEARS)**				
<45	34	36.75 (5–67)	0.278	0.598
≥45	46	34.74 (13–84)		
**SEX**				
Male	43	38.65 (6–84)	0.865	0.352
Female	37	33.77 (5–67)		
**DIFFERENTIATION**				
Good	58	40.51 (6–84)	15.378	0.000
Moderate and Poor	22	24.32 (5–42)		
**TUMOR SIZE**				
<5 cm	24	39.58 (12–62)	0.999	0.318
≥5 cm	56	33.93 (5–84)		
**AJCC STAGE**				
I/ II	61	39.56 (6–84)	17.666	0.000
III/IV	19	22.93 (5–61)		
**ENNEKING STAGE**				
I	55	40.85 (6–84)	17.025	0.000
II/ III	25	25.01 (5–61)		
**METASTASIS**				
No	69	37.69 (6–84)	5.459	0.019
Yes	11	25.74 (5–61)		
**INVASION**				
No	13	76.08 (18–84)	6.134	0.013
Yes	67	57.82 (5–67)		
**DAXX**				
–	39	42.13 (16–84)	6.327	0.012
+	41	30.41 (5–67)		
**DRD3**				
–	40	40.85 (16–84)	5.570	0.018
+	40	30.87 (5–62)		
**DISC1**				
–	34	37.34 (17–63)	0.697	0.404
+	46	34.09 (5–84)		

On multivariable Cox regression analysis, moderate and poor differentiation (*P* = 0.006), AJCC stage III/IV (*P* = 0.013), Enneking stage II/III (*P* = 0.016), metastasis (*P* = 0.033), invasion (*P* = 0.011), positive DAXX staining (*P* = 0.033), and positive DRD3 staining (*P* = 0.025) correlated negatively with the postoperative survival rate and positively with mortality. Expression of DAXX and DRD3, but not DISC1, independently predicted CS (*P* < 0.05; Table 4).

**Table 4:** Associations of DAXX, DRD3, and DISC1 expression and clinicopathological characteristics with overall survival in patients with chondrosarcoma, as determined by multivariable Cox regression.

**Characteristics**	***P***	**RR**	**95% CI**	
			**Lower**	**Upper**
Age (<45 vs.≥45)	0.447	1.270	0.686	2.350
Sex (Male vs. Female)	0.848	0.940	0.498	1.774
Differentiation (Good vs. Moderate and Poor)	0.006	2.067	1.222	3.495
Tumor size (<5 cm vs. ≥5 cm)	0.363	0.662	0.272	1.611
Metastasis (No vs. Yes)	0.033	6.284	1.162	33.973
Invasion (No vs. Yes)	0.011	3.834	1.362	10.793
AJCC stage (I/II vs. III/IV)	0.013	3.219	1.279	8.102
Enneking stage (I vs. II/III)	0.016	3.080	1.231	7.708
DAXX (– vs. +)	0.033	2.697	1.082	6.722
DRD3 (– vs. +)	0.025	2.563	1.125	5.837
DISC1 (– vs. +)	0.052	0.427	0.181	1.009

In the competing-risks model, tumor differentiation (*P* = 0.005), metastasis (*P* = 0.014), invasion (*P* = 0.028), AJCC stage (*P* = 0.003), Enneking stage (*P* = 0.036), and DAXX (*P* = 0.039), and DRD3 (*P* = 0.019) expression were independent predictors of death from CS. No variable was an independent predictor of death of other causes (Table 5).

**Table 5:** Competing risks regression models for 80 patients with CS.

**Characteristics**	**Death from CS**		**Death of other causes**	
	**HR (95%CI)**	***P***	**HR (95%CI)**	***P***
**AGE**				
<45 vs.≥45	1.13 (0.58–2.21)	0.716	0.49 (0.08-3.03)	0.442
**SEX**				
Male vs. Female	1.19 (0.61–2.30)	0.615	6.60 (0.74–59.11)	0.092
**DIFFERENTIATION**				
Good vs. Moderate and Poor	2.94 (1.37–6.27)	0.005	1.55 (0.25–9.51)	0.636
**TUMOR SIZE**				
<5 cm vs. ≥5 cm	1.32 (0.63–2.75)	0.463	0.65 (0.10-4.06)	0.644
**METASTASIS**				
No vs. Yes	3.30 (1.27–8.59)	0.014	1.76 (0.19–15.93)	0.617
**INVASION**				
No vs. Yes	1.94 (1.08–3.65)	0.028	0.43 (0.17–1.12)	0.086
**AJCC STAGE**				
I/II vs. III/IV	3.49 (1.55–7.85)	0.003	0.74 (0.08–6.66)	0.786
**ENNEKING STAGE**				
I vs. II/III	2.42 (1.06–5.53)	0.036	0.92 (0.10–8.28)	0.938
**DAXX**				
– vs. +	2.11 (1.04–4.28)	0.039	1.33 (0.21–8.24)	0.761
**DRD3**				
– vs. +	2.35 (1.15–4.82)	0.019	0.59 (0.09–3.68)	0.570
**DISC1**				
– vs. +	1.54 (0.78–3.03)	0.214	0.43 (0.07–2.67)	0.362

**Figure 3:**
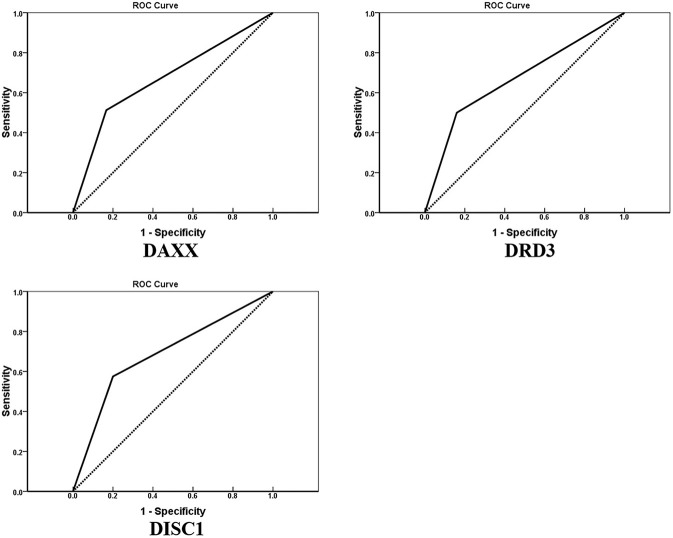
Receiver operating characteristic curves for DAXX, DRD3, and DISC1 protein expression in 80 patients with chondrosarcoma (January 2011–June 2015). Areas under the curves are 0.673, 0.670, and 0.688, respectively.

AUCs for DAXX, DRD3, and DISC1 were 0.673 (95% CI, 0.557–0.788; *P* = 0.010), 0.670 (95% CI, 0.556–0.784; *P* = 0.011), and 0.688 (95% CI, 0.573–0.802; *P* = 0.005), respectively (Figure 3).

## Discussion

Data on the association between mental health and cancer are conflicting. On one hand, mental conditions have been reported to play important roles in cancer promotion and development (Cohen et al., 2007; Barik et al., 2013). Mental disorders such as schizophrenia have been found to be associated strongly with many cancers; for example, individuals with schizophrenia are at greater risk of developing lung cancer (Kisely et al., 2013), and some studies have identified a modestly elevated risk of breast cancer in these individuals (Vancampfort et al., 2015). Moreover, patients with schizophrenia appear to be more likely to die of cancer once that cancer is diagnosed (Irwin et al., 2014). Possible explanations for these findings include delayed diagnosis and lack of access to screening, which lead to more advanced staging at the time of diagnosis, and reduced access to or use of appropriate treatments after diagnosis. For example, people with depression are less likely to receive routine cancer screening (Carney and Jones, 2006), which may result in more advanced disease at presentation (Liang et al., 2017). On the other hand, some studies have documented a reduced incidence of cancer in patients with dementia and schizophrenia (Attner et al., 2010; Bushe and Hodgson, 2010; Kisely et al., 2013). Biological theories for the reduced incidence in patients with schizophrenia include a protective effect of excess dopamine, enhanced natural killer cell activity, increased apoptosis, modulation by antipsychotic drugs of cytochrome enzymes involved in mutagen activation and elimination, and neonatal vitamin D deficiency (Bushe and Hodgson, 2010; Wang et al., 2011). Although the cancer incidence is no higher in psychiatric patients than in the general population, the former are more likely to have metastases at diagnosis and less likely to receive specialized interventions (Kisely et al., 2013).

Given this contradictory evidence for the relationship between mental health and cancer, we investigated the role of mental health in CS, which to our knowledge has not been examined previously. We found that the expression of DAXX, DRD3, and DISC1 was significantly greater in CS tissue than in benign OC tissue. In addition, the expression of these mental health–related proteins was associated with CS severity, progression, and poor prognosis. These findings suggest that DAXX, DRD3, and DISC1 may be useful biomarkers for this malignancy.

We found significantly higher percentages of positive DAXX expression in tumors with poorer differentiation, later AJCC and Enneking stages, and metastasis. We also demonstrated that DAXX is an independent predictor of CS. Similar functions of DAXX have been reported previously. In prostate cancer, strong DAXX expression was associated strongly with a high Gleason grade, advanced pT stage, increased cell proliferation index, and early prostate-specific antigen recurrence (Tsourlakis et al., 2013). Chou et al. (2018) also found that DAXX and ATRX losses were mutually exclusive and associated with other adverse clinicopathological variables and poor survival in patients with pNETs. Furthermore, 4-Hydroxy nonene aldehyde can promote DAXX localization to the cytoplasm and inhibition of apoptosis by blocking the apoptosis signal–regulating kinase 1 pathway (Sharma et al., 2008), associated with the progression of cervical cancer (Tang et al., 2014). Thus, the results of the present study and previous studies suggest that DAXX can serve as a biomarker of CS development and progression.

In the present study, the expression of DRD3 correlated positively with poor differentiation, late AJCC and Enneking stages, and metastasis and negatively with the mean survival time of patients with CS. Thus, DRD3 could serve as a biomarker of CS progression and predictor of its prognosis. Our findings are in accordance with those of some previous studies (Yin et al., 2015), but D2-like receptors have been found to have diverse effects in different cancers. These contradictory conclusions, including ours, may reflect heterogeneity among cancer types (Malchenko et al., 2012). In addition, D2-like receptors, including DRD3, have inconsistent functions (Wang et al., 2019). The relationship between the immune and nervous systems is significant in oncogenesis; neurotransmitters, including dopamine, affect immune cells in many diseases (Reiche et al., 2004; Ganguly et al., 2010), leading to changes in the numbers of T cells, which play a key role in the immune system. T cells reduce the viability of tumor cells via secretion of the cytokine TNF-α, but the overexpression of D2-like receptor genes down-regulates this effect (Jiang et al., 2007). Thus, the association between DRD3 expression and CS progression observed in this study may be a result of the down-regulation of anti–tumor cell cytokines.

Although DISC1 expression was not associated with the mean postoperative survival time in this study, it (like DAXX and DRD3) was related strongly to CS progression, indicating its utility as a biomarker. This finding is in agreement with Wang et al. (2017), who reported that DISC1 promotes non-small cell lung cancer growth, probably through the GSK3β/β-catenin signaling pathway. Moreover, in glioblastoma, knockdown of DISC1 was found to significantly inhibit cell proliferation, migration, and invasion *in vitro* and *in vivo* (Gao et al., 2016). This effect of DISC1 was likely related to mitochondrial dynamics, in part via the down-regulation of Drp1. The mechanisms underlying the role of DISC1 in cancer have been well studied. DISC1 is an important cell proliferation regulator that positively modulates Wnt signaling via the inhibition of GSK3β catalytic activity and activation of β-catenin (Brandon et al., 2009). GSK3β/β-catenin signaling pathway has been found to be associated with the pathogenesis of cancers (Wan et al., 2015; Wang et al., 2017). Another important mechanism of DISC1 is its regulation of the cAMP-signaling pathway, which has been demonstrated to be associated strongly with cancer pathogenesis and promotion (Simko et al., 2017). In the cAMP/PKA pathway, DISC1 interacts with several key molecules that are important in the pathogenesis and progression of cancers (Palorini et al., 2016), including a D2 dopamine receptor that suppresses cAMP production. The association of DISC1 expression with CS progression is supported by these findings.

Unexpectedly, expression of the mental health–related proteins was not associated with CS invasion in this study, even though invasion into surrounding tissue and vasculature is an important step in cancer metastasis. DAXX and DISC1 have been associated with tumor invasion in previous studies (Gao et al., 2016; Lin et al., 2016); to our knowledge, DRD3 has not. We also detected significant positive correlations between the expression of DAXX and DRD3, DAXX and DISC1, and DRD3 and DISC1, suggesting that these proteins mutually regulate each other or that their expression is regulated through the same pathway. However, the underlying mechanism remains unclear, and further studies are required to understand it.

The results of this study should be interpreted with caution, as the study has some limitations. The sample size was small and the study was performed at only two academic medical centers. In addition, the findings were not validated in an independent patient cohort. However, we believe that the results of this study provide a useful reference for further research.

In conclusion, in the present study, we found that the mental health–related proteins DAXX, DRD3, and DISC1 were expressed strongly in CS tissue. Overexpression was observed in samples showing moderate and poor differentiation, AJCC stage III/IV, Enneking stage II/III, and metastasis. Moreover, DAXX and DRD3 expression was associated significantly with shorter mean survival times of patients with CS. These results suggest that the DAXX, DRD3, and DISC1 proteins could serve as biomarkers of CS progression and predictors of its prognosis.

## Data Availability Statement

The datasets generated for this study are available on request to the corresponding author.

## Ethics Statement

The studies involving human participants were reviewed and approved by Medical Ethics Committee of the Second Xiangya Hospital, Central South University. Written informed consent to participate in this study was provided by the participants' legal guardian/next of kin.

## Author Contributions

LH and XS analyzed the patient data and performed the IHC staining. RC and ZW participated statistical analysis. ZL wrote the manuscript. ZY revised the manuscript. All authors read and approved the final manuscript.

### Conflict of Interest

The authors declare that the research was conducted in the absence of any commercial or financial relationships that could be construed as a potential conflict of interest.

## Abbreviations

ALT: alternative lengthening of telomeres
cAMP: cyclic adenosine monophosphate
CS: chondrosarcoma
DAXX: death domain–associated protein
DISC1: Disrupted-in-schizophrenia-1
DRD3: dopamine receptor D3
GSK3β: glycogen synthase kinase 3 beta
IHC: immunohistochemistry
OC: osteochondroma
pNET: pancreatic neuroendocrine tumor
PH: proportional hazards.
